# Alzheimer’s disease: targeting the peripheral circulation

**DOI:** 10.1186/s13024-023-00594-8

**Published:** 2023-01-11

**Authors:** Zhi-Hao Liu, Yan-Jiang Wang, Xian-Le Bu

**Affiliations:** 1 Department of Neurology and Centre for Clinical Neuroscience, Daping Hospital, Third Military Medical University, Chongqing, China; 2 Chongqing Key Laboratory of Ageing and Brain Diseases, Chongqing, China; 3 Department of Neurology, The First Affiliated Hospital of Wenzhou Medical University, Wenzhou, China; 4 Institute of Brain and Intelligence, Third Military Medical University, Chongqing, China; 5 State Key Laboratory of Trauma, Burns and Combined Injury, Third Military Medical University, Chongqing, China; 6 Center for Excellence in Brain Science and Intelligence Technology, Chinese Academy of Sciences, Shanghai, China

**Keywords:** Alzheimer’s disease, Blood exchange, Aβ clearance, Systemic factors

Alzheimer’s disease (AD) is the most common neurodegenerative disease and is characterized by progressive memory loss and learning impairment. Dysfunction of amyloid-beta (Aβ) clearance is believed to be the main cause of Aβ accumulation in sporadic AD, which accounts for 99% of all AD cases [1]. Ageing, the most significant risk factor for AD, has a profound impact on the peripheral system, including inflammation, immune cell skewing, increased levels of ageing-related factors, and reduced levels of youth-related factors [2]. The impact of these systemic changes on the development of AD has been seriously underestimated in the past. A substantial number of parabiosis and plasma exchange studies have indicated that numerous systemic factors mediate brain ageing, brain Aβ pathology and cognitive decline through blood–brain barrier, perivascular glymphatics and meningeal lymphatics [2]. Age-related changes in human haematopoiesis cause reduced regenerative capacity and immune dysfunction during ageing, and the impaired energy metabolism of aged peripheral myeloid cells could lead to cognitive decline [3]. Recently, a large prospective study showed that peripheral immunity is associated with AD risk [4]. Of note, we found that approximately 40%–60% of brain-derived Aβ is cleared by the peripheral organs and tissues, and enhancement of peripheral Aβ clearance can decrease brain Aβ deposition and prevent AD pathogenesis [5, 6]. Therefore, AD may be a systemic disease with dysfunction of Aβ clearance beyond the brain, and we need to understand AD pathogenesis and develop therapies from a systemic perspective.

Recently, a study by Akihiko Urayama et al. that was published in *Molecular Psychiatry* showed a significant reduction in brain Aβ burden and memory improvement in transgenic AD mice after exchanging whole blood from 5-week-old wild-type (Wt) mice [7]. In the first experiment, they performed a 300 μL blood exchange in 3-month-old Tg2576 mice and continued the treatment once a month until 13 months of age. After multiple treatments, 40–60% of the original blood was replaced by donor blood. They found that the Aβ plaque number and percentage area in the cerebral cortex and hippocampus were 50% to 75.9% lower in the blood exchange group than in the sham-operated and untreated transgenic mice. The Barnes’ maze test showed that both short- and long-term memory were improved to the level of Wt mice in Tg2576 mice after blood exchange treatments. Then, the researchers extended the treatment period for an additional 4 months and found that the Aβ plaque number and burden in 17-month-old AD mice subjected to blood exchange were similar to those in 13-month-old sham mice. These results indicate that this treatment could prevent brain Aβ deposition and persistently reduce the rates of Aβ plaque growth. To further investigate whether this treatment could reduce brain Aβ after extensive Aβ plaque formation in the brain, the researchers initiated the treatment at 13 months of age and evaluated the brain Aβ burden and spatial memory at 17 months of age. They found that the Tg2576 mice subjected to blood exchange showed a reduced Aβ burden in both the cerebral cortex and hippocampus compared to the sham controls. The spatial memory of the Tg2576 mice subjected to blood exchange was also improved to the level of Wt mice. Taken together, this study suggests that the peripheral circulation plays a crucial role in AD pathogenesis and that continuous whole blood exchange has both preventive and treatment potential for AD.

While these findings are encouraging for therapeutic benefit of blood exchange, there are some limitations to this study that could be addressed in future research. First, only the Barnes maze test was used to assess cognitive function in this study, so the cognitive evaluation was incomplete. Second, aged Tg2576 mice had severe cerebral amyloid angiopathy-dependent cerebrovascular dysfunction, which could confound the interpretation of blood exchange of Aβ between the circulation and the brain. Third, the Tg2576 model mice used in Urayama et al.’s study overexpressed human amyloid precursor protein (APP) containing Swedish mutation under the control of the hamster prion protein promoter. As nonneural tissues such as heart, intestine, circulating leukocytes, and other peripheral cells also express the prion protein gene promotor, APP will be expressed in peripheral tissues as well as in the brain in Tg2576 mice. Thus, the decreased brain Aβ burden in the Tg2576 mice subjected to blood exchange may result from the elimination of peripherally produced Aβ. Lastly, there was a lack of investigation of specific mechanisms of action associated with whole blood exchange beyond enhanced Aβ clearance mediated by young blood.

Increasing evidence suggests that there is a dynamic interchange of brain-derived pathological proteins between the brain and peripheral blood. Blood Aβ and p-tau are abnormal in synchrony with cerebrospinal fluid values, and blood-based AD biomarkers show great potential for the early diagnosis, prognosis, or monitoring of disease progression [8]. In particular, intracerebral Aβ can efflux into the peripheral system and be cleared by the liver, kidney, and blood [5, 6]. Deficits in Aβ clearance by the peripheral system also contribute to the development of AD [5, 6], revealing the substantial contribution of the peripheral system to the clearance of brain-derived Aβ. We also demonstrated that the ability of the blood to clear Aβ decreased with ageing and AD progression [9]. Thus, young blood exchange could enhance Aβ catabolism in the periphery and reduce the brain Aβ burden. In addition to Aβ, brain-derived pathological tau can also flow to the blood and be cleared in the periphery, which contributes to tau clearance from the brain [10]. Whether blood exchange can also enhance tau catabolism in the periphery requires further study. Moreover, blood cell-produced Aβ can enter the brain and induce brain Aβ deposition, and the reduction in peripheral Aβ production can alleviate brain AD-type pathologies and behavioural deficits [11]. Therefore, the decreased brain Aβ burden in the Tg2576 mice subjected to blood exchange may result from the elimination of peripherally produced Aβ. It demonstrated that 4 weeks of young plasma administration could restore synaptic function and cognition without reducing the brain Aβ burden in AD mice [12]. Recent studies revealed that liver-produced peripheral apolipoprotein E4 (ApoE4) could impair synaptic plasticity [13, 14] and affect cerebrovascular function [14]. Plasma from young apoE3 mice (but not apoE4 mice) could improve cognitive function and the neurovascular unit in aged Wt mice [14]. Based on this evidence, we speculate that whole blood exchange may protect against AD by increasing the levels of protective factors and decreasing the levels of harmful factors (Fig. 1). However, the exact mechanism by which systemic factors reduce the brain Aβ burden and improve memory is presently unknown. Of note, whole blood replacement with blood from young mice is the most direct method of rejuvenating peripheral immune cells. Thus, the whole blood replacement performed in Urayama et al.’s research may have exerted an anti-AD effect by alleviating peripheral immune ageing (Fig. 1). Recently, a phase 2b/3 trial showed that plasma exchange with albumin replacement improved memory, language abilities, and quality of life in patients with mild-to-moderate AD [15]. Consequently, blood exchange might be an effective intervention strategy for the prevention and treatment of AD (Fig. 1).

**Fig. 1:**
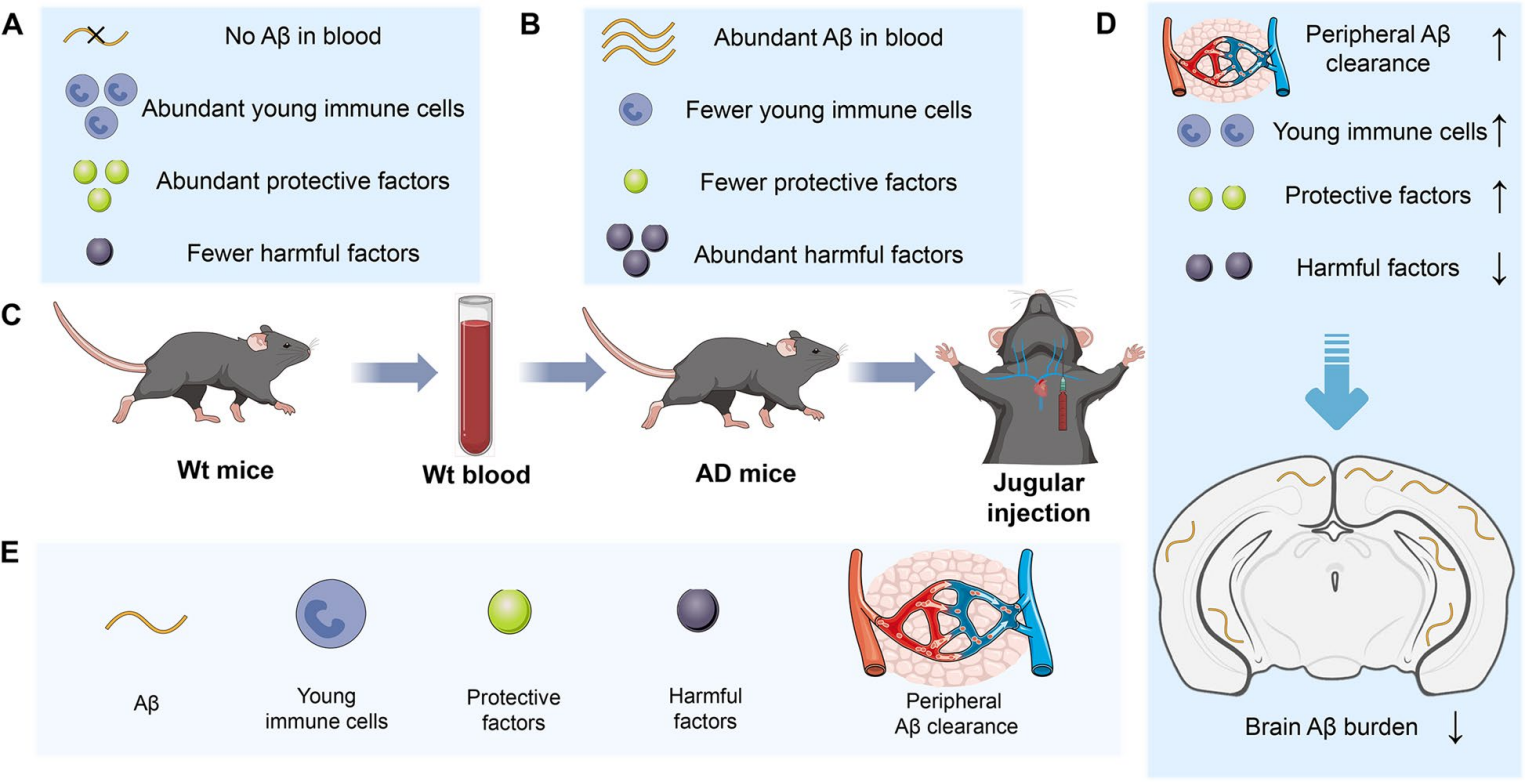
The potential mechanisms of whole blood exchange as an intervention for Alzheimer’s disease. **A**) Young wild-type (Wt) mice have abundant young immune cells and protective factors but fewer harmful factors and no Aβ in the blood. **B**) Elderly transgenic mice with Alzheimer’s disease (AD) have fewer young immune cells and protective factors and abundant harmful factors and Aβ in the blood. **C**) Schematic diagram of whole blood exchange. **D**) Transplantation of blood from young Wt mice into elderly AD mice could enhance the peripheral clearance of Aβ, increase the number of young immune cells and protective factors, reduce the number of harmful factors, and ultimately alleviate brain Aβ deposition. **E**) Key for the symbols used in this figure

In summary, Urayama et al.’s paper is one of the several recent studies highlighting the role of the peripheral circulation in the pathogenesis of AD, which opens new avenues to develop disease-modifying interventions for AD and raises several interesting questions. As numerous systemic factors in the peripheral circulation are involved in AD, the precise mechanism by which blood exchange generates a therapeutic benefit in AD mice needs to be investigated further, which could assist in the development of more effective, specific, and practical intervention strategies for AD. In addition, it remains unclear how systemic factors in the peripheral circulation are regulated, especially how different ApoE isoforms regulate systemic factors and mediate AD progression. It is imperative to elucidate the roles of these factors in different stages of AD in primate models and humans using multiomics techniques in the future.

## Data Availability

Not applicable.

## Abbreviations

AD: Alzheimer’s disease
Aβ: Amyloid-beta
APP: Amyloid precursor protein
ApoE: Apolipoprotein E
CNS: Central nervous system
Wt: Wild-type

## References

[1] Selkoe DJ, Hardy J (2016). The amyloid hypothesis of Alzheimer's disease at 25 years. EMBO Mol Med.

[2] Pluvinage JV, Wyss-Coray T (2020). Systemic factors as mediators of brain homeostasis, ageing and neurodegeneration. Nat Rev Neurosci.

[3] Minhas PS, Latif-Hernandez A, McReynolds MR, Durairaj AS, Wang Q, Rubin A, Joshi AU, He JQ, Gauba E, Liu L (2021). Restoring metabolism of myeloid cells reverses cognitive decline in ageing. Nature.

[4] Zhang YR, Wang JJ, Chen SF, Wang HF, Li YZ, Ou YN, Huang SY, Chen SD, Cheng W, Feng JF (2022). Peripheral immunity is associated with the risk of incident dementia. Mol Psychiatry.

[5] Xiang Y, Bu XL, Liu YH, Zhu C, Shen LL, Jiao SS, Zhu XY, Giunta B, Tan J, Song WH (2015). Physiological amyloid-beta clearance in the periphery and its therapeutic potential for Alzheimer's disease. Acta Neuropathol.

[6] Chen SH, He CY, Shen YY, Zeng GH, Tian DY, Cheng Y, Xu MY, Fan DY, Tan CR, Shi AY (2022). Polysaccharide krestin prevents Alzheimer's disease-type pathology and cognitive deficits by enhancing monocyte amyloid-beta processing. Neurosci Bull.

[7] Urayama A, Moreno-Gonzalez I, Morales-Scheihing D, Kharat V, Pritzkow S, Soto C: Preventive and therapeutic reduction of amyloid deposition and behavioral impairments in a model of Alzheimer's disease by whole blood exchange. Mol Psychiatry 2022. 10.1038/s41380-022-01679-4 PMC10601825 35835859

[8] Huang S, Wang YJ, Guo J (2022). Biofluid biomarkers of Alzheimer's disease: progress, problems, and perspectives. Neurosci Bull.

[9] Chen SH, Tian DY, Shen YY, Cheng Y, Fan DY, Sun HL, He CY, Sun PY, Bu XL, Zeng F (2020). Amyloid-beta uptake by blood monocytes is reduced with ageing and Alzheimer's disease. Transl Psychiatry.

[10] Wang J, Jin WS, Bu XL, Zeng F, Huang ZL, Li WW, Shen LL, Zhuang ZQ, Fang Y, Sun BL (2018). Physiological clearance of tau in the periphery and its therapeutic potential for tauopathies. Acta Neuropathol.

[11] Sun HL, Chen SH, Yu ZY, Cheng Y, Tian DY, Fan DY, He CY, Wang J, Sun PY, Chen Y (2021). Blood cell-produced amyloid-beta induces cerebral Alzheimer-type pathologies and behavioral deficits. Mol Psychiatry.

[12] Middeldorp J, Lehallier B, Villeda SA, Miedema SS, Evans E, Czirr E, Zhang H, Luo J, Stan T, Mosher KI (2016). Preclinical assessment of young blood plasma for Alzheimer Disease. JAMA Neurol.

[13] Giannisis A, Patra K, Edlund AK, Nieto LA, Benedicto-Gras J, Moussaud S, de la Rosa A, Twohig D, Bengtsson T, Fu Y (2022). Brain integrity is altered by hepatic APOE epsilon4 in humanized-liver mice. Mol Psychiatry.

[14] Liu CC, Zhao J, Fu Y, Inoue Y, Ren Y, Chen Y, Doss SV, Shue F, Jeevaratnam S, Bastea L (2022). Peripheral apoE4 enhances Alzheimer's pathology and impairs cognition by compromising cerebrovascular function. Nat Neurosci.

[15] Boada M, Lopez OL, Olazaran J, Nunez L, Pfeffer M, Puente O, Pinol-Ripoll G, Gamez JE, Anaya F, Kiprov D (2022). Neuropsychological, neuropsychiatric, and quality-of-life assessments in Alzheimer's disease patients treated with plasma exchange with albumin replacement from the randomized AMBAR study. Alzheimers Dement.

